# Perspectives on delivering safe and equitable trauma-focused intimate partner violence interventions via virtual means: A qualitative study during COVID-19 pandemic

**DOI:** 10.1186/s12889-022-14224-3

**Published:** 2022-10-04

**Authors:** Winta Ghidei, Stephanie Montesanti, Lana Wells, Peter H. Silverstone

**Affiliations:** 1 School of Public Health, University of Alberta, 3-266 Edmonton Clinic Health Academy 11405-87 Ave, T6G 1C9 Edmonton, Canada; 2 School of Public Health, Centre for Healthy Communities, University of Alberta, Edmonton, Canada; 3 Department of Psychiatry, University of Alberta, Edmonton, Canada; 4 Brenda Strafford Chair, Prevention of Domestic Violence, University of Calgary, Calgary, Canada

**Keywords:** Intimate Partner violence, Equity, Safety, Virtual-delivery of interventions, Trauma-focused, Underserved populations

## Abstract

**Background:**

The COVID-19 pandemic has been linked with increased rates of intimate partner violence (IPV) and associated experiences of compounded trauma. The emergence of this global pandemic and the public health measures introduced to limit its transmission necessitated the need for virtually delivered interventions to support continuity of care and access to interventions for individuals affected by IPV throughout the crisis. With the rapid shift to virtual delivery, understanding the barriers to accessing virtually delivering trauma-focused IPV interventions to these individuals was missed. This study aimed to qualitatively describe the challenges experienced by service providers with delivering virtually delivered IPV services that are safe, equitable, and accessible for their diverse clients during the COVID-19 pandemic.

**Methods:**

The study involved semi-structured interviews with 24 service providers within the anti-violence sector in Alberta, Canada working with and serving individuals affected by IPV. The interviews focused on the perspectives and experiences of the providers as an indirect source of information about virtual delivery of IPV interventions for a diverse range of individuals affected by IPV. Interview transcripts were analyzed using inductive thematic analysis.

**Results:**

Findings in our study show the concepts of equity and safety are more complex for individuals affected by IPV, especially those who are socially disadvantaged. Service providers acknowledged pre-existing systemic and institutional barriers faced by underserved individuals impact their access to IPV interventions more generally. The COVID-19 pandemic further compounded these pre-existing challenges and hindered virtual access to IPV interventions. Service providers also highlighted the pandemic exacerbated structural vulnerabilities already experienced by underserved populations, which intensified the barriers they face in seeking help, and reduced their ability to receive safe and equitable interventions virtually.

**Conclusion:**

The findings from this qualitative research identified key determining factors for delivering safe, equitable, and accessible virtually delivered intervention for a diverse range of populations. To ensure virtual interventions are safe and equitable it is necessary for service providers to acknowledge and attend to underlying systemic and institutional barriers including discrimination and social exclusion. There is also a need for a collaborative commitment from multiple levels of the social, health, and political systems.

## Background

Intimate Partner Violence (IPV) is a global health problem [1] defined as a behavior, including financial and coercive control, by a current or former intimate partner that causes physical, sexual, or psychological harm. IPV can include physical aggression, psychological and emotional abuse, and sexual coercion either alone or in combination [1]. Although individuals of all gender identities experience IPV, women and girls are most likely to experience severe IPV and associated adverse outcomes, including femicide [1]. Globally, one in three women are exposed to violence within intimate partner relationships [2]. In Canada, IPV is the leading cause of serious injury and the second leading cause of death among reproductive age women [3].

The COVID-19 pandemic has highlighted the worsening social and economic inequalities that have emerged in recent decades across the globe and has aggravated the vulnerability of certain population groups. Research evidence shows that there is an interrelationship between the social determinants of health (SDOH) and IPV [4]. When the basic determinants of health such as, housing stability and income are not met, women or girls are more susceptible to interpersonal violence, and likewise, the occurrence of interpersonal violence among women and girls can further impact on those determinants. IPV severity is shaped by factors such as financial stain resulting in increased stress among individuals and families [5, 6]. Rates of IPV-related police reports and calls to violence hotlines increased by 30% during the pandemic in Canada [7]. In a recent report by the Ending Violence Association of Canada (EVAC), 82% of frontline service providers surveyed within the anti-violence sectors in Canada observed an increase in the severity of violence experienced by their clients [8].

IPV is a form of trauma with significant short-term and long-term psychological consequences that range from stress, frustration, anger, and decreased social function to severe depression, anxiety, post-traumatic stress disorder (PTSD), and suicidality [9, 10]. Individuals affected by IPV during the COVID-19 pandemic are struggling with complex trauma as a result of the violence and pandemic-related mental distress [11]. Evidence shows there is a direct relationship between the length of quarantine and level of negative psychological effects (e.g., depression, stress, anxiety) [12, 13]. For some survivors of IPV who have left the abusive relationship, the stay-at-home orders implemented during the first and second wave of the pandemic in Canada triggered recollection of past traumatic experiences and heightened their anxiety [8]. IPV survivors were also cut off from community and support networks. In the EVAC report referenced above [8], service providers caring for IPV survivors identified three main concerns for survivors seeking support during the pandemic: (1) clients who depend on face-to-face interactions faced setbacks in their healing process and mental health; (2) delays in family court proceedings impacted some clients’ safety from violence; and (3) survivors were not seeking out care, which had adverse mental health outcomes given they were struggling with complex trauma.

While no specific group in the Canadian population is exempt from experiences of IPV, research to date has found that different populations may have different experiences of IPV, in terms of its prevalence, characteristics, and impacts [14]. Even though police reports of IPV are low overall, the prevalence of IPV in both police-reported and self-reported metrics in Canada is significantly lower for underserved populations [15, 16]. These populations include Indigenous peoples, immigrants, refugees, racial and ethnic diverse groups, official language minorities, LGBTQ2S + individuals, individuals living with disabilities, those experiencing homelessness or precariously housed, sex trade workers, and individuals of low socioeconomic status [17]. Understanding the varied experiences between groups is crucial to the delivery of appropriate services, programs, or prevention strategies related to IPV. Furthermore, structural barriers in Canada such as the ambiguity of the criminal justice process and racist interactions with the police deter Indigenous, Black and racialized individuals from reporting experiences of abuse to authorities [16].

### Barriers to accessing appropriate interventions and supports among individuals affected by IPV

#### An Overburden Anti-Violence Sector

While the demand for anti-violence services has increased over the years in Canada, funding for anti-violence services has not kept pace, contributing to increased pressure on anti-violence workers, and unmet needs among individuals affected by IPV. A decade of austerity measures not only reduced direct funding for organizations within the anti-violence sector, but also increased demand on the sector as a whole, by reducing service provision. Unfortunately, the COVID-19 pandemic struck at a time when IPV services in Canada had been substantially cut, or underfunded, for many years. Multiple funding cuts to the sector since the early 2000s have resulted in the needs of individuals demanding IPV support going unmet [18–20]. Therefore, the lack of available core sustainable funding structure, including the inequitable distribution of government funding, changes in policy, and high staff turnover within the anti-violence sector, has had a significant impact on service providers and others working with individuals affected by IPV and weakened the capacity of the sector overall [18–21].

Moreover, specialist IPV services for underserved communities are in a particularly precarious position. Research by Women’s Shelters Canada found that staff turnover and burnout are a major challenge [22]. During the pandemic, EVA Canada surveyed Gender-Based Violence (GBV) organizations and discovered that 81% of frontline workers experienced additional workplace stress due to the pandemic [8]. Additionally, 84% of workers reported health and safety concerns while doing their jobs during the pandemic. The constraints faced by the anti-violence sector has compromised the quality and safety of services and therapeutic interventions delivered to survivors and individuals experiencing IPV. As a result, individuals seeking support fall through the cracks.

#### Help-seeking barriers among survivors

Studies show that the rate of help-seeking for specialized IPV support among underserved populations from both medical and social providers is low [10, 23]. Factors such as unemployment, low educational status, economic dependence and experiences of violence have been identified as obstacles to disclosure of violence and seeking support [24]. The barriers are further compounded for individuals with intersecting social identities (i.e., those who belong to more than one socially disadvantaged populations) [25]. Existing evidence suggests that Indigenous peoples and ethnic minority women often avoid seeking support from healthcare providers because of feelings of mistrust and fear toward service providers [26, 27].

Systemic inequalities underpin how institutions practice, what services are available, and how services are provided and received by these individuals [16, 28, 29]. Fear of the system and a lack of services are barriers for women and gender diverse people who are trying to get the help they need to leave an abusive situation. In Canada, one in five women has reported experiencing racism and culturally insensitive and inferior quality of care within healthcare systems and mainstream social services [28]. These barriers discourage Indigenous, Black and other racialized women from seeking help when experiencing IPV [28, 29]. For these women, the decision to seek or access help is also influenced by several intersecting factors. These include: (i) cultural norms and patriarchal ideologies which “stigmatize, blame and shame women from exposing IPV or seeking help” [30]; (ii) structural barriers, such as the ambiguity of the criminal justice process, racist interactions with the police and court system, spousal sponsorship policies, legal status, and fear of deportation [16]; and (iii) culturally insensitive care within the healthcare system and mainstream social services [28, 29]. Women with disabilities also face physical and structural barriers to help seeking which include impaired mobility, complete dependency on the perpetrator, and lack of accessible services [31]. Systemic inequalities in gender, race, class and disability were heightened during the COVID-19 pandemic and posed a greater risk of exposure to IPV.

## Study context

### Service Delivery Adaptations during COVID-19

During the COVID-19 pandemic in Canada, the public-health measures introduced to limit the transmission of the COVID-19 virus have meant that most services (including mental health and specific trauma-focused interventions) abruptly pivoted to virtual delivery [32]. During the stay-at-home mandate, virtual delivery of interventions and services such as web-services, tele-counseling, telepsychiatry, e-mental health programs, and violence hotlines became vital resources for individuals affected by IPV [2]. Virtual delivery of interventions incorporates the use of technology to provide communication, education, intervention, or service between a provider and a client [33–35]. However, virtual delivery of interventions and services was not widely adopted by service providers within the anti-violence sector prior to the COVID-19 pandemic and, when used, was restricted to remote areas with limited access to in-person services [33]. Since the pandemic, however, the vast majority of those providing services and interventions to individuals affected by IPV moved to providing services via virtual platforms. However, the shift to providing services virtually, although necessary – and beneficial to many – has exacerbated the gap and barriers with delivering interventions to individuals affected by IPV [36].

It has been reported that the risks to a women’s safety increases when she uses app-based interventions that can easily be accessed by her perpetrator [37]. Inequitable access to these virtually delivered IPV interventions is also a key barrier. For example, some individuals may be struggling with unstable or unavailable internet connections, or they may not be able to afford the required devices to receive treatment or support virtually [8, 38, 39]. The speed at which virtually delivered interventions and services are available to individuals affected by IPV has also raised concerns for service providers within the anti-violence sector. Anecdotal evidence shows, in the province of Alberta, demand for trauma counselling services related to experiences of sexual violence increased during the pandemic, resulting in an average wait-time of 18-months to see a trauma counsellor. Additional barriers to uptake of virtually delivered trauma interventions can include issues concerning confidentiality and privacy and not being comfortable with receiving interventions commonly delivered face-to-face over phone or video [8].

There is limited data on how these challenges have impacted individuals affected by IPV in accessing trauma-focused interventions during the pandemic. Trauma-focused interventions are specific approaches to therapy that recognize and “emphasize how the traumatic experience impacts an individual’s mental, behavioral, emotional, physical, and spiritual well-being” [40]. Further knowledge in this area is fundamental to enable safe, equitable, and accessible approaches to virtual delivery of IPV-related interventions during the pandemic and beyond. Safety in the virtual delivery of IPV interventions encompass the physical, emotional, and cultural safety of the individual accessing services via videoconferencing, telephone or online. These include having a structured place where the individuals’ physical and emotional safety is respected and where they feel empowered “to seek, share, and obtain information, access services, express themselves, enhance psychosocial wellbeing, and more fully realize their rights.” [41]. Equity in the virtual delivery of IPV interventions refers to fairness and justice in the availability and distribution of these interventions to a wide range of population groups, and addresses practices that systematically marginalize and stigmatize entire population groups [42]. Finally, accessibility refers to the ability to attain affordable, client-centered, culturally appropriate IPV interventions and services virtually [43]. To this end, this study aimed to qualitatively explain, from the perspectives of service providers in Alberta, the challenges with accessing virtually delivered interventions that are safe, equitable, and accessible for a diverse range of individuals affected by IPV during the COVID-19 pandemic. This study is guided by intersectional feminist theory which highlight the importance of challenging inequities based on sexism, racism, colonialism, class, and other social factors [44, 45].

## Methods

Semi-structured interviews were conducted with 24 service providers within the health and anti-violence sectors working with and serving individuals affected by IPV in Alberta (Table 1). An interview guide was co-developed by all authors and interviews were conducted by the lead author. The interview guide covered a wide range of topics relevant to the virtual delivery of IPV interventions during the pandemic, and to better understand the facilitators or barriers that individuals affected by IPV might experience in accessing virtually delivered IPV interventions. Some of the interview questions include the following:

- What is your current role within your department or organization? How long have you been in this role?
- What has been your experience in delivering virtual interventions to the patient or clientele population you serve? Probe: Can you describe your experience with virtual interventions during COVID-19?
- What do you see as the opportunities and challenges to delivering virtual interventions to individuals affected by IPV? Probe: issues with confidentiality and privacy? Probe: issues with logistics, cost, engagement of users?
- How can virtual interventions that incorporate trauma-focused treatment for individuals affected by IPV be adapted or tailored across a range of diverse populations (e.g., immigrants and refugees, Indigenous, rural/remote communities)?
- How would you compare virtual delivery of interventions to a traditional in-person approach in responding to the needs of diverse individuals affected by IPV?

The semi-structured interviews also explored the providers’ needs and challenges in delivering virtual interventions. The findings pertaining to service providers’ experiences has been published elsewhere [46]. All interviews were conducted over a two-month period via telephone or videoconference and lasted approximately one hour in duration. The participants were given detailed information both verbally and written about the aims of the study and the voluntary nature of their participation. All participants provided written and verbal consent to participate. Participant interviews continued until data saturation was reached. The study protocol was reviewed for its adherence to ethical guidelines by a Research Ethics Board at the University of Alberta (REB # Pro00101547).

### Participant recruitment

A purposive maximum variation sampling technique [47], which involves deliberately selecting individuals who fit the criteria for the study and represent diverse populations, was used to recruit participants. Thus, participants from various organizations and geographical locations serving individuals affected by IPV from diverse social, economic, and cultural backgrounds were invited to participate. Interview participants were recruited from existing relationships among the research team and community partners. Research team (SM, LW) have strong relationships with stakeholders within the anti-violence sector in Alberta. These include stakeholders at Sagesse Domestic Violence Prevention Society and IMPACT in Alberta. IMPACT is a provincial collective impact initiative to eradicate domestic violence and sexual violence in Alberta [48]. The initiative brings together a network of over 400 systems and organizations that represent thousands of anti-violence workers “to address shared issues, enhance services and supports across Alberta and identify opportunities for large-scale change” [48]. The study was promoted through IMPACT and email invitations were sent to potential participants asking them if they would like to be interviewed for the study. The email explained what the study is about and the voluntary nature of their participation. Participants in the study included directors, managers, psychologists, counselors, mental health support workers, clinical directors from sexual assault services, family physicians and outreach workers and advocates from the social services sector, health, and school divisions across Alberta (Table 1). The majority of participants were female (n = 17). Of these female participants eleven were white and six were racialized individuals (Indigenous, Black and South Asian). Of the male participants (n = 4), three were white and one was a racialized individual. Most of the participants (n = 15) have worked within the anti-violence sector for less than ten years (Table 1).

### Data Management and Analysis

All the interviews were audio recorded and transcribed with verbal consent of the informants. Interviews were analyzed using QSR NVivo 12 software, to facilitate data management and to enhance the systematic organization and examination of the data. Qualitative data analysis was undertaken by the first and second authors (WG and SM). We focused our analysis on the perspectives and experiences of service providers as an indirect source of information about virtual delivery of IPV interventions. Interview transcripts and field notes were analyzed using an inductive thematic analysis process following the six steps outlined by Braun and Clarke [49]. First the researchers familiarized themselves with the data by reading and re-reading the transcripts and getting a sense of what the data is showing. Then interview transcripts were coded and further organized into categories. Next, the codes/categories were reviewed, and patterns were identified. This resulted in the generation of major themes. The first author re-read all the interview transcripts to ensure the themes accurately represent the data and the themes were updated accordingly. Once consensus was reached in the theme development stage, names and definitions were developed that clearly and succinctly describe what each theme means and represents. Finally, the results were used to write up this article. All identifiable information was removed to ensure participants were not recognized through their quotes.

An intersectional feminist perspective requires researchers to undertake a continual process of self-reflection and become attuned to how power is used throughout the construction of the research [50–52]. Self-reflection entails interrogating the identity of the researcher (gender, race, class, etc.) as well as their relationship with their participants and its effect on how they conduct the research project [51, 53]. As part of self-reflection process, the researchers wrote memos throughout the research to examine how their own biases shaped their approach to data collection, interpretation of findings, and decision-making. They engaged in weekly debriefing sessions and continuously questioned how their own identities shaped the research inquiries and how they receive and interpret the information from the participants [54].

**Table 1:** Participant Profile

Number of Participants	
Organizations Type (n) Non-Profit Organizations Community-Based Agencies Women’s Shelters Primary Care Networks (PCNs) Networks and Collaboratives Addressing Gender-Based Violence School Division	N = 24 9 5 3 3 2 2
Participant Roles Executive Director Clinical Director Program Director Consultant Program or Project Manager/Coordinator Physician Registered provisional psychologist Outreach counselor Clinical supervisor	N = 24 5 3 3 3 4 2 2 1 1
City/Town Edmonton Calgary Spruce Grove, Stony Plain and Parkland County Fort McMurray High River Lethbridge Medicine Hat Red Deer	N = 24 9 6 4 1 1 1 1 1
Number of years worked within the anti-violence sector 2–5 years 5–10 years 10–15 years 15–20 years 20–25 years 25–30 years	N = 24 9 6 4 1 2 2

Table 1 outlines the profiles of service providers who participated in this study.

## Results

Our findings highlight three interrelated factors that posed challenges to delivering and accessing trauma-focused IPV interventions that are accessible, equitable, and safe for a diverse range of individuals during the COVID-19 pandemic. The following themes emerged from service provider interviews: (i) Acknowledging pre-existing systemic and institutional barriers that impact delivery of and access to any IPV intervention, (ii) How the COVID-19 pandemic changed help-seeking behaviours for IPV interventions, and (iii) Difficulties ensuring client safety in the virtual environment when providing care or treatment for clients. These three factors are discussed in more detail below.

### Acknowledging pre-existing systemic and institutional barriers that impact delivery of and access to any IPV intervention

Service providers described the barriers their clients face when accessing IPV-related interventions. These barriers are compounded for underserved and socially disadvantaged groups who experience multiple oppressions related to race, class, ability, sexuality, gender, socio-economic status, and where they live. Service providers described the inequities in access to IPV interventions:

“*I think that there is inequity in our system. I think for sure, ethnoculturally diverse communities, black people, indigenous people, people of color, our services are not equitable for them*.” [P 3]

“*At one of the last counts we had over 80 or around 80 languages spoken here, and unfortunately most of the time our services are in English*.” [P10].

“*Because of stigma and systematic racism we see that newcomers and first-generation immigrants are hesitant to reach out for support*” [P19].

Furthermore, participants highlighted there is a gap in compassionate, client-centered and trauma-informed care for socially disadvantaged populations, instead existing IPV interventions are generalized and generic:

*“The current model is very program-centric, that is that the client has to fit the program, and not the program figuring out how to fit the client*…*you almost have to be a systems navigator to figure out all the different agencies that are in the community, and what service is going to be able to work with that particular client around a particular need. And very often women get referred to services and then come back and say, well, they said they could not help me, I don’t fit their mandate or do don’t fit their program*.” [P11]

“*And so I think from a systemic level we are forcing people into a box that is not the box that they need...and I think that if we as the social workers, and as the sector, really listened to them, we would see that many of the gaps that we traditionally identify actually are not about gaps in service, but about gaps in how we view what we think the client experience is, and what we think the client needs, versus what the client is actually telling us, and what the stats actually tell us*.” [P20]

Other providers also identified a gap in interventions designed for gender diverse individuals, persons with disabilities, and a lack of culturally appropriate treatment services for immigrant populations. For underserved populations with intersecting identities these challenges are even greater. Some service providers indicated that there is a gap in the provision of intersectional trauma-focused interventions within the anti-violence sector. One provider explained instead of providing holistic approaches to addressing their clients’ intersecting needs, their organization focuses on “*either a cultural perspective, or a gender perspective, or an age demographic perspective,”* this provider added, *“those [identities] get split out versus having a service that can deal with all of those moving pieces*” [P1].

Nonetheless, some service providers explain these gaps in the provision of client-centered interventions are a byproduct of the funding constraints experienced by the anti-violence sector. One provider stated, “*I think funding is always a gap*.” [P19] and continued to explain how lack of sufficient funding complicates the ability to hire more staff, to provide additional training and to ensure the availability of culturally appropriate services. As previously described, anti-violence organizations face significant funding challenges leading to patchwork supports, disconnected systems, resource scarcity, and a workforce that is poorly compensated and at-risk of burning out. The challenges with long wait lists and the inability to provide timely services to individuals affected by IPV was highlighted by participants in this study. Moreover, participants shared the pandemic has depleted their resources and they expressed concern that they may not be able to sustain current outreach programs.

A number of service providers also shared that this lack of funding resources contributed to a lack of coordination, collaboration and transparency among agencies within the anti-violence sector due to competing needs for further funding. As one service provider describes,

“*I would say there is lack of coordination and effective service delivery integrated together…I think people struggle with working together sometimes because you’re afraid that if you work together and more collaboratively, are you going to lose your autonomy or you’re fighting for funding with the person you are collaborating with…I think people do not wake up in the morning, saying, I do not want people to be served appropriately. I think it’s about our systems get in the way of supporting each other. The health system refers to us constantly but don’t fund us.*” [P 15]

Another provider further explained despite the good intentions of the organizations and providers within the sector, they have not been able to work together towards a common goal as a result of the previously explained constrains: “*it’s not that there’s not enough good people in the world, it’s just that they do not see one another’s mandates as linked, especially when it comes to complex social issues in our region*.” [P8] The lack of sufficient funding, and the resulting lack of collaboration within the anti-violence sector impacts how individuals affected by IPV can seek, access and use IPV interventions and services. This is illustrated in the following quotes from providers:

“*Systems can be self-serving and funding limited. So when that happens I don’t think that necessarily families are given all of the information to be able to make a decision for themselves at all times*.” [P1]

“*[IPV survivors] probably find their way to an agency because somebody told them to call, and hopefully that agenda steers them through that and gets them to the right one, but I would say that it’s not very trauma-informed*.” [P15]

In summary, the lack of sufficient resources due to the existing funding structures reinforce competition and lack of collaboration within the anti-violence sector which complicates the existing multi-layered barriers individuals face when accessing IPV interventions and services.

### How the COVID-19 pandemic changed help-seeking approaches for IPV interventions

The COVID-19 pandemic further exacerbated the previously discussed systemic- and institutional-level challenges in accessing trauma-focused IPV interventions. Despite experiencing complex trauma and intensified levels of violence during the pandemic, initially individuals affected by IPV did not reach for help or supports. Providers shared that multiple barriers kept this group of clients from reaching out for help. The barriers included being trapped with an abusive partner, being disconnected from informal support systems, and worrying about contracting the COVID-19 virus itself. Informal and formal support services are vital to individuals affected by IPV; however, during the pandemic these supports were not easily accessible to these individuals. For example, interview participants shared their experiences and perspectives on the impact of isolation during the pandemic on their clients’ help-seeking approaches from informal and formal supports as follows:

“[For] s*urvivors there is an increased risk in that isolation of further victimization because they’re not interacting with as many people outside the home*.” [P23]

“*They simply did not feel comfortable in receiving calls, talking, so there has been an impact in their ability to access [support services]*.” [P7]

Even when some individuals wanted to escape and run to shelters, they were worried about the uncertainties of acquiring the COVID-19 virus. Thus, individuals affected by IPV were forced to make difficult choices between risking their and their families’ health or staying in a potentially injurious and traumatizing household. Service providers shared some of their clients’ experiences as follows:

“[*They had] to make tough decisions about exposing [themselves] to what might be the virus out there, or staying in your home and be safe from the virus not safe from the violence*.”[P3]

“*I think that there is the real fear that exposure to COVID could possibly be potentially worse than what they’re experiencing…we had clients trying to contemplate whether or not the possibility of contracting COVID is worth the risk of staying home with the perpetrator*.” [P7]

Unfortunately, in some cases when individuals affected by IPV tried to reach out for support services, the essential services were not always accessible or available to them due to pandemic-related restrictions and challenges. One provider summarizes some of their clients’ experiences as,

“*I think that COVID has added so much pressure and so much stress. Court dates were stopped. Some services were stopped in terms of our ability to serve everybody. We used to have groups of ten to 12 folks. Now we have groups in person of six. Even Zoom groups were at eight because we did a little gauging on what would be an ideal number for Zoom. And so the accessibility of the services in a time of greater need, I would say, has been cut down, adding to that stress.*” [P 21]

Additionally, service providers commented in detail on the impact of the digital divide as a barrier to accessing virtually delivered IPV interventions during the pandemic. The digital divide refers to the inequitable access to internet and technology, socio-economic barriers, language barriers, low literacy levels, and limited access to virtually delivered interventions and services [55]. Financial barriers and poverty play a major role in an individual’s ability to access virtually delivered interventions as explained by one provider, “*financially and economically the victim may not have, frankly, a cell phone, or whatever to be able to access virtual care, potentially*.” [P4] A majority of the service providers also highlighted the challenges their clients face in their inability to afford and access laptops, cell phones, desktop computers, stable internet, or even internet at all. In some cases, even when an organization is able to provide their clients with the equipment and technology they need, the clients may not be able to access stable internet connection due to their geographical location. One provider explains, “*for some of our remote and rural communities, even if we could send a client a tablet to be able to connect with us online, they need effective data or Wi-Fi, or whatever it is. And some rural and remote places in Alberta definitely don’t have that*.” [P3].

Similar challenges with internet access were also shared by other service providers as following:

“*I think the big barrier right now is that we don’t consider internet connectivity as a fundamental right or as a utility. I think that’s really major that in this day and age and particularly in a pandemic environment, connectivity is literally a lifeline for some people. So, that’s a really serious barrier*.” [P9]

“*How do we get these services to folks if they don’t have that technology, if they don’t have stable Wi-Fi or any Wi-Fi to access? So, I think it exacerbated that ability to reach out and to connect with others because, yes, all these services are still available but to access them, that just creates a bigger gap*.” [P10]

Some participants insightfully spoke about how digital exclusion experienced during the pandemic is a reflection of pre-existing inequities with access to services. In addition to dealing with financial barriers, IPV survivors from socially disadvantaged populations (e.g., Indigenous, immigrant and homeless individuals) experience an additional layer of inequity when accessing interventions and services virtually. The following quotes from providers describe some of these barriers and challenges:

“*As [an Indigenous] woman I believe that the Canadian healthcare system is systematically racist and I think that a lot of that would be lost in that translation or in that transition [to virtual delivery], or it would just exacerbate it*” [P10].

“*Issues of digital equity are deeply rooted, connected and systemic. To understand if virtual care is acceptable and the factors that influence the use of virtual services, we need ask what bars people from using the services they do have access to?*” [P3]

“*A lot of the clients that are served in Alberta – 50%, 55% are Indigenous women and children. And in their homes and on reserve and even off reserve, whether or not they have the laptops, the internet, Wi-Fi, to be able to access services electronically – I think that would disadvantage them; and probably newcomers [too]. So, I think there would be some disparity in terms of access to services if it went virtual. Like, if that was to become the method of counselling*.” [P11]

Therefore, individuals affected by IPV from socially disadvantaged populations faced transecting barriers in accessing IPV-related interventions during the pandemic. In addition to the previously identified barriers, individuals with disability also face specific challenges when accessing and using virtually delivered services. Some interview participants shared some of these challenges as follows:

“*I’ve worked with the deaf and hard of hearing community, and I know that oftentimes they connect with therapists through face time, but there would be extra barriers during this time period just with technological pieces and being able to access interpreters, that kind of thing.*” [P23]

“*some people could not be served on Zoom. We have a client with quite profound hearing loss who lip-read. Zoom for them is out...we don’t have any facilitators who know a lot of ASL*.” [P21]

Therefore, the COVID-19 pandemic and related restrictions, financial strain and the digital divide layered the complexities in seeking and accessing support or therapeutic interventions for socially disadvantaged individuals.

### Difficulties in ensuring safety in the virtual environment for individuals affected by IPV

As previously indicated, the public health stay-at-home orders during the pandemic isolated individuals affected by IPV which resulted in a more complex set of issues including increased exposure to abusive partners while dealing with barriers to safely seeking for or accessing support services virtually. At the same time, ensuring safety of individuals affected by IPV when they are virtually accessing IPV-related interventions and services was also a complex issue for service providers. In this study, service providers identified safety concerns in relation to security and physical safety when accessing services virtually, emotional safety when receiving services virtually, and cultural safety of available IPV-related interventions.

Service providers in our study highlighted staying at home with an abusive partner who is also dealing with pandemic related stressors exacerbated the risk of experiencing violence for their clients. A service provider shared,

*“...if you’re in that vulnerable relationship, and let’s say they’re having more stressors like financial, or they lost their job and then they can’t get their unhealthy coping strategies, like maybe their alcohol or marijuana or whatever it is, we know where they’re going to lash out*.” [P5]

Interview participants also highlighted the severity of violence experienced by their clients during this time was much worse than the providers have ever seen before. A director of an outreach center shared some examples as following: “*we’re seeing a lot more strangulation…we had one that was an attempted murder. So, the seriousness and the levels of domestic violence has increased*” [P 14]. Another service provider attributed these experiences of severe violence to the lack of viable options to safely getaway during the lock downs: “*for some of them well they were home alone with their abuser. So, some of the abuse is more extensive than it would have been if they had avenues of escape like going out, going to school, other eyes watching*” [P 24].

When and if individuals affected by IPV wanted to reach out for help or support, interview participants indicated, they may not have private and safe physical place where they can access virtual interventions, especially if they are quarantined with their perpetrator. For individuals affected by IPV, being caught in the act of talking to a provider could exacerbate the risk of violence and thus could hinder them from accessing any virtual services at all. A service provider shared how some clients could not access or seek for help virtually due to fear of their abusive partner discovering that they were speaking to a professional:

“*I had a case where a lady had finally opened up about her situation at home, and I had to call her, and we couldn’t even talk, because she was scared to even say anything, she didn’t know where her spouse or partner was, she was still hoping just to be able to come see me and be able to talk without having to fear what if I say something, or what if he hears just the slightest word, that makes me in trouble or that I’ve done something wrong*.” [P5]

Similarly, other service providers described how it was difficult to reach out to and provide services virtually to their clients due to concerns for their safety and worry about creating any further harm for their client:

“*I think there is a safety and a confidentiality perspective that you can’t always get in your home. That you maybe don’t actually have access. Even if you can have technology access, that if you’re living with somebody who is using violence or could be using violence, that accessing programming is unrealistic. And I do think that community is harder to form in a virtual context*.” [P20]

“*I think the main thing is the safety of the client, if the person is there how to communicate with them, unless they are out of the house...either the perpetrator is there or the children are there who can then tell the perpetrator what the mother was doing. So that communication piece, that’s a challenge*.” [P19]

“*The privacy and confidentiality, which connects in some cases to the safety and security, that it just might not be safe in the home for women to electronically connect to their counsellors or support, because it could easily be discovered or overheard*.” [P11]

To address this, providers suggested setting up safety plans such as safe words between clients and providers, where if the client uses the safe word, the session ends, and they will contact each other in a different predetermined approach. Interview participants shared some examples where providers, staff and organizational leaders had to be creative in how to approach this in a virtual environment. For instance, some providers shared examples of how they developed safety plans with their clients:

“*If she showed something on the computer, [the provider and the client] had a sign between them that only the two of them know, that if she displayed that, then that was the therapist’s cue that something was going down and she needed help right away*.” [P14]

*“I would mostly check-in, pretend to be a tele-marketer and have a code phrase, and they would respond yes, no, goodbye. And that would give us a clue as to whether they were safe in their home.*” [P7]

Furthermore, in the event that a client is able to access virtual support or treatment in the absence of their abusive partner, there are still privacy risks that could also jeopardize their safety when accessing IPV interventions virtually. Providers spoke about the importance of modifying their organizations’ policies and procedures to protect clients’ privacy in the provision of interventions virtually and ensuring security and privacy safeguards when using different virtual platforms.

Service providers also described that it was difficult to implement trauma-focused interventions when there is a risk of triggering and/or re-traumatizing their clients who are receiving treatment from their home – a typical place where the violence has occurred. One service provider explains: “*I think some people in their places, their living spaces, it’s going to be quite triggering and re-traumatizing. Everything around them could be a trigger*.” [P 17] This concern was echoed by other providers who explained if their clients are triggered during an in-person session, they were better equipped to manage and support them, while feeling unsure how to manage this in a virtual setting:

*“I am hesitant [about virtual services], especially the clientele that I serve, who are coming in for years of domestic violence, I have to be sure that if I am providing trauma care, what kind of support that person has. Often these females have young children, they are living by themselves, I don’t know if I’m doing something, and then she gets triggered. There are young babies at home. How can you manage that?”* [P19]

“*… within our groups, there’s real vulnerability and safety. You create the safety, and then you can have the vulnerability for them to share with each other. It’s totally a different interface sharing in person than it is on a screen*.” [P21]

In addition to addressing psychological and emotional safety, the importance of incorporating culturally safe approaches to healing trauma when providing IPV interventions virtually was widely discussed by service providers. Some service providers explained that some underserved populations such as, Indigenous peoples, immigrants, and newcomers may not feel safe to share their experiences in a virtual environment due to being misunderstood or that virtually-delivered interventions are not culturally safe for them. This is described in the following quote from a service provider:

“*Then of course the cultural aspect is huge, trying to feel that cultural safety when they are talking...so of course they want the person who is sitting across from them, listening to their story, want them not to be judged because they are immigrants, or they are from a certain ethnic background”* [P19].

Other providers shared similar perspectives and highlighted the importance of client-centered care by engaging clients and addressing their unique needs in the virtual delivery of IPV interventions. A service provider who shared this viewpoint emphasized the significance of asking underserved populations to see what works for them as follows:

“*There may be communities who say for whatever reason this does not work for our community, there is something in our cultural make up that does not fit this. I think we have to find that out first, as opposed to pretending that we know what is best for everybody else…our Indigenous brothers and sisters, what about this work, does it work for them? And let’s talk to new immigrants and say does this work, is this right, are you more comforted or less comforted?*” [P18]

Moreover, service providers expressed the importance of incorporating cultural safety and addressing their clients’ experiences of trauma holistically (i.e., addressing structural violence, and generational and historical trauma). One provider gave an example of how their organization promotes holistic interventions which is grounded in the understanding of their client’s context, individual barriers and the root causes of their trauma. This provider further explains, *“their trauma is not just trauma of [IPV]. If you have an indigenous woman waiting into your office, it’s also trauma of colonialism and identity and there is all of these intersecting pieces.”* [P 3]. Other service providers also added,

“*I think I would suggest that trauma focused treatment and client-centered treatment, my definition of what it is means that it has to be specialized and adapted. And so, I think that by creating a co-shared understanding with our client about their context and their world is exactly how we do that work.*” [P3]

*“when you’re seeing a different culture and the way they talk to each other or the way they handle stress, we can’t just say, well, you can’t do that here. You really have to approach it in a very easy and kind of non-judgmental way with an education piece...there’s so many different cultures, and there’s different ideas and ideologies of them...The biggest thing, a lot, would be that validation piece, and being able to let them know that no one can tell you what to do, no one can tell you when to do it...no one can tell you when you’re supposed to leave or what you’re supposed to do. You have to be totally comfortable and feel safe with whatever your choices are. I think that’s a big piece of that virtual care, no matter what kind of culture it is.*” [P5]

“*You have to understand that different cultures will have different perspectives about...And I honor that. So, I think making sure staff are culturally competent and aware, and respect people’s different cultures, faiths and traditions is really important*.” [P15]

Additionally, having services and interventions delivered by providers of similar cultural background or who speak the same language as the clientele population was also described to facilitate delivery of culturally safe and client-centred services and interventions. This was described for Indigenous and newcomer clients as follows:

*“…having available a therapist who has some experience working with Indigenous communities or understands some of the spirituality or narrative practice..*.” [P2]

“*For individuals that struggle with English it’s very difficult to provide virtual services, especially if there isn’t a webcam. I wouldn’t say they struggle with English, rather we [as providers] struggle with understanding them. It’s a bit challenging, and so maybe we look at how do we hire providers who can provide virtual interventions in their mother tongue*.” [P18]

Service providers in this study emphasized the importance of providing culturally safe, trauma-informed, client-centered, and contextually specific interventions to a diverse group of individuals affected by IPV during the pandemic and beyond.

## Discussion

**Fig. 1:**
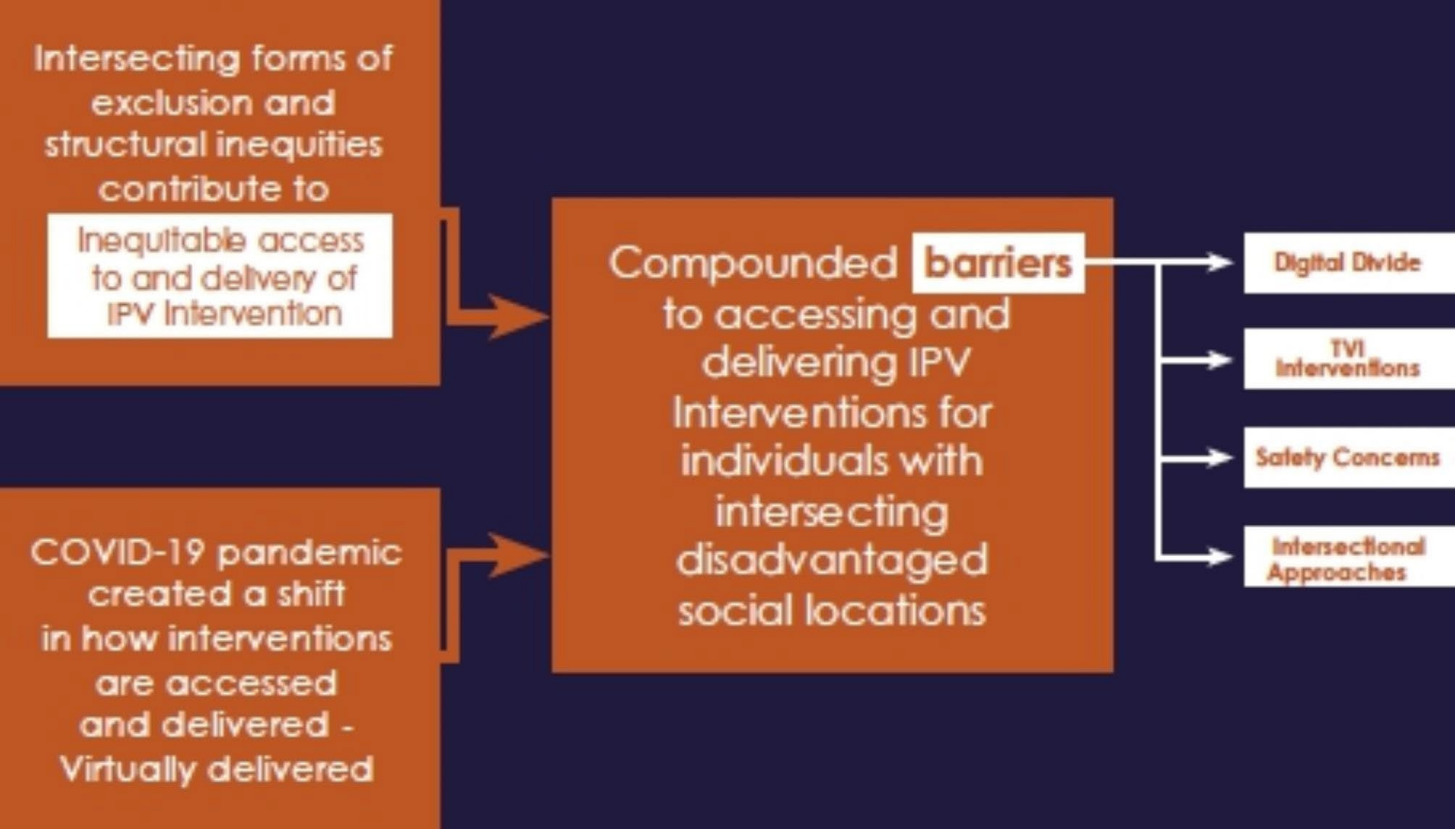
The compounded effects of social exclusion, structural inequities and the impact of the global pandemic exacerbated barriers faced by individuals affected by IPV in accessing support services and interventions

Our study examined from the perspective of service providers how trauma-focused virtual IPV interventions can be safe, equitable, and accessible for range of diverse individuals affected by IPV, particularly for those who are socially disadvantaged. Studies show the shift to virtual delivery of interventions provided several opportunities for individuals who may not otherwise be able to receive these services, including providing access to individuals residing in remote locations, offering convenience in terms of saving time, removing transportation barriers, and allowing clients to maintain connection with care providers during the pandemic [22]. However, findings in our study show that the concepts of equity and safety are more complex for individuals affected by IPV, and the barriers they face in accessing IPV interventions and services during a pandemic were further compounded. In summary, the COVID-19 pandemic exacerbated pre-existing inequities within the anti-violence sector that serve individuals affected by IPV. The pandemic further complicated the ability for these individuals to feel physically and emotionally safe, exacerbated vulnerabilities experienced by underserved populations, and intensified the barriers they face in seeking help and accessing safe and equitable interventions virtually (see Fig. 1).

As previously highlighted, the anti-violence sector was weakened by lack of sustainable funding and resources and thus unprepared to accommodate the sudden influx of IPV support needs from a wide range of individuals during the pandemic. With many organizations at capacity prior to the pandemic and with increasing demand during the pandemic, organizations will require additional funding to increase service capacity to address the increase even after the pandemic is resolved. For instance, any wait time for counselling interventions presents considerable challenges for people who have experienced complex trauma and who are struggling to cope, and longer wait times are even more detrimental to the healing process. Similarly, researchers in the US also reported that the pandemic exacerbated pre-existing inequities and gaps in services within the anti-violence sector [56], including limited resources that could not support the needs of increased demand and needs of individuals affected by IPV seeking support during the pandemic [56].

In response to the increased demand faced by the anti-violence sector across Canada during the pandemic, the federal government provided $50 million to women’s shelters, sexual-assault centers, and other organizations providing support and services to women experiencing violence [19]. An additional $350 million was then provided for community organizations serving individuals experiencing violence [19]. Although organizations within the sector appreciate this support, some anti-violence workers highlight “it will take more to overcome the legacy of years of underfunding in the sector” [19]. Service providers in our study also shared their hope for continued sustainable funding for the anti-violence sector. In addition to these funding related barriers, providers in our study highlighted the lack of coordination and collaboration within and across sectors, making it even more difficult for individuals affected by IPV to access support services and resources. It is essential for organizations that serve IPV survivors to form alliances within and across different sectors to better serve vulnerable population groups.

During the pandemic, individuals affected by IPV also had to navigate accessing care and treatment through virtual modalities. Recent studies have highlighted the multiple barriers that vulnerable and socially disadvantaged individuals experience with virtual delivery of interventions. Recent research highlights the consequences of inequitable access to virtual care, characterized as the ‘digital divide’ [55]. The ‘digital divide’ is shaped by access (does the virtual intervention reach clients?) and uptake (are clients using the virtual intervention?). Service providers in our study described multiple barriers to accessing digital technologies experienced by underserved populations, who are at a greater risk of IPV during the pandemic. Interview participants noted these barriers exist for individuals residing in rural and remote communities, individuals experiencing homelessness, Indigenous peoples, immigrants and refugees, individuals with disabilities as well as those experiencing financial strain. These findings support recent studies which report individuals who reside in rural settings with limited access to internet, those who cannot afford technology and individuals with disability are facing further disparities during this new shift to virtual delivery of interventions and services [33, 57, 58]. Access to stable and reliable internet services remain a challenge for many Canadians [59]. Thus, there is an urgent need to tackle the digital divide by funding broadband infrastructure and increasing digital literacy for a wide range of diverse individuals affected by IPV. Further research is needed to examine how digital exclusion is experienced by diverse population groups, and across intersecting factors of gender, sex, age, geography, disability, race, ethnicity and culture.

Furthermore, service providers in our study highlighted the challenges their clients faced in accessing virtually delivered services remotely throughout the pandemic, emphasizing the lack of safe and private space to attend virtual sessions. This is in line with recent literature that report the stay-at-home orders in the early months of the pandemic have exacerbated the safety concerns for individuals affected by IPV and their ability to safely access interventions in person or virtually [39]. Some common concerns shared by service providers in our study and in other academic reports include an abusive partner may overhear their conversations with a provider, or may not allow them to speak in private, or some abusers may use control and monitoring measures such as recording phone calls that could put these individuals at greater risk if they seek help virtually [39]. This complicates the experiences of individuals affected by IPV immensely: it increased their risk of experiencing more frequent and more severe abuse, while hindering them from safely accessing support services. Thus, our study findings suggest safety planning can potentially be a way to protect clients’ safety when accessing services virtually. Safety planning is defined as a dialogic process that informs and supports individuals at risk of IPV by identifying behaviors they can adopt to increase safety and decrease exposure to violence for themselves and their family [60]. Pre-pandemic safety planning has been commonly used in response to or in order to prevent experiences of IPV [61]. A recent study investigated the need for modified safety planning strategies for individuals affected by IPV during the COVID-19 pandemic within a Canadian context; and identified 19 IPV safety planning strategies that were considered safe to use during the pandemic by IPV survivors and service providers [62]. However, the study also outlined the strategies are context and situation specific (i.e., may not be generalizable for all women experiencing IPV) [62]. Therefore, there is still limited evidence of its effectiveness in pandemic context when public health restrictions are in place.

For individuals affected by IPV, the concept of safety in itself is also a complex phenomenon. In addition to safety from direct experience of abuse, diverse individuals affected by IPV also require cultural safety that will ensure the available resources are meaningful to their needs. For example, providers in our study identified gaps in culturally safe approaches that address the service essentials of individuals with intersecting social disadvantages who are affected by IPV. Other studies also report racialized populations face barriers to accessing virtually delivered interventions due to a lack of culturally acceptable and appropriate virtual tools [8]. Sabri et al., [63] made similar observations in a study they conducted with immigrant IPV survivors in the US, and they added this population group was “less comfortable and less able to engage effectively with virtual resources” due to the lack of culturally safe approaches. Therefore, ensuring physical, emotional, and cultural safety for individuals affected by IPV in virtually delivered interventions is key.

Service providers in this study also highlighted their clients’ need to feel safe from re-traumatization when they are receiving trauma-focused interventions virtually. The prevalence and profound health impacts of trauma and violence have been well documented in the literature [64, 65]. Although providers in our study indicated they provide comprehensive trauma-focused interventions to their clients, it was clear from their responses that a more holistic trauma-and-violence informed approach is needed to better serve underserved populations in Alberta. Trauma- and violence-informed (TVI) approaches build on trauma-informed approaches and bring attention to the broader social conditions impacting people’s health; ongoing violence, including institutional violence; discrimination and harmful approaches embedded systems, structures and social norms [66, 67]. Thus, TVI approaches centre on understanding the context in which peoples’ challenges are experienced and recognize how this intersects with IPV, structural violence, inequity and trauma; including trauma attributed to the devastating effects of colonialism and racism. Structural violence refers to how societies, including their institutions and policies, are organized in ways that cause harm to some people [42]. Therefore, TVI approaches work to create safe and accessible services for people impacted by trauma and violence by focusing on the experiences of trauma holistically, preventing re-traumatization, and empowering individuals affected by IPV [68]. As a result, TVI interventions are key to addressing the mental health and other needs of individuals affected by IPV from diverse population groups.

As previously stated, the intersecting barriers faced by diverse individuals affected by IPV in accessing safe, and equitable virtually delivered interventions result from structural inequities that shape experiences with discrimination, exclusion, and mistreatment. Our findings show that during the COVID-19 pandemic, individuals with heighted vulnerabilities who were affected by IPV had to cope with complex trauma and more frequent and severe violence, while also navigating issues of systemic discrimination and inequality preventing them from accessing trauma-focused IPV interventions or services. Similarly, other researchers in Canada explained the pandemic increased the existing gaps in availability of services for underserved populations while also intensifying experiences of racism and discrimination [69]. These findings are in line with similar reports from countries in the northern hemisphere [8, 56, 63, 69]. Researchers claim the pandemic brought into light the “historic, systemic, and structural inequalities at the intersection of racial and ethnic minority status, occupation, and class.” [69, 70]. As a result, some scholars recommend that we view the current global crisis as a ‘syndemic’ [69, 70]. The concept of syndemics (the interaction or cooperation of two or more epidemics) acknowledge the existence of epidemics and pandemics in the context of pre-existing social and health conditions [71]. Such a viewpoint would go beyond outlining the barriers faced by underserved individuals affected by IPV during a single global crisis towards illuminating the underlying sources of these intersecting forms of inequities [69, 70]. That is to explain why these individuals are more vulnerable in the first place [69, 70]. Therefore, addressing barriers to accessing safe and equitable virtually delivered IPV-related interventions will require unpacking and confronting the root causes of these barriers in the first place.

To summarize, the delivery of safe and equitable IPV-related interventions will necessitate consistent and collaborative effort from governing bodies, organizations and service providers serving individuals affected by IPV. At a systemic level, addressing racism and discrimination requires an understanding of the root causes of inequity. Some examples of such initiative include developing policies that are guided by a decolonizing and anti-racism lens; and meaningfully engaging diverse voices and perspectives from Indigenous and other racialized populations [69]. At the same time, it is essential for the federal and provincial governments to promote sustainable funding to address the digital divide and assure greater funding for the anti-violence sector to employ providers from diverse backgrounds who can relate to the clients’ cultural needs, hire language interpreters, and provide training opportunities for delivering trauma- and violence-informed (TVI) interventions. Providers in our study also shared their recommendations for mitigating the social and economic inequities their clients face in seeking for and accessing virtually delivered interventions. The providers emphasized the need for community and stakeholder involvement in the design and implementation of virtually delivered interventions, respecting local values, and addressing the core needs of clients to ensure the interventions are equitable and accessible by all. Scholars also recommend the virtual delivery of interventions to be mindful of the values and needs of the individuals affected by IPV, conducted respectfully and in collaboration with local community-based organizations, and connect to local resources [34, 72]. Sherwin et al., [72] specifically stated, “if virtual care is to remain the focus of how care is delivered at scale, now is the time to define the value proposition for [clients], systems, payors and regulators.”

## Strengths and limitations

Our study has some strengths and limitations. To our knowledge, this paper is the first to report on the determinants of safe, and equitable interventions for IPV survivors in Alberta, Canada. Second, our findings add valuable information to the growing body of literature on the complex and multi-dimensional issues faced by individuals affected by IPV in seeking for and virtually accessing IPV-related interventions and services during a pandemic. Despite these strengths, there are a few limitations to this study. Although data saturation was reached in this study, most of the included participants were providers at a managerial position, thus we are missing first-hand experiences of frontline providers in this sector; therefore, some other dimensions of the phenomenon may have been overlooked. In addition, the experiences of individuals affected by IPV with accessing virtually delivered interventions was reported from the perspective of service providers, thus their clients’ voices are not included in this paper. However, for the next phase of our research, our team is engaging individuals affected by IPV in a photovoice project to better understand their experiences, challenges and barriers with accessing virtually delivered interventions and services during the COVID-19 pandemic. Finally, our study only focused on how the virtual delivery of interventions affected individuals experiencing IPV, at-risk of IPV, or survivors of, IPV but it did not address the experiences of perpetrators seeking or accessing virtually delivered interventions for support.

## Conclusion

The current global pandemic significantly contributed to the increased rates and severity of IPV experienced by diverse individuals, and it further illuminated pre-existing social exclusions and inequities faced by underserved population groups. For these individuals, accessing and using IPV-related interventions safely has become more complex as a result of the digital divide characterized by inequitable access to safe virtual platforms because they are being controlled by their abusers, struggling with unstable or unavailable internet connections, unable to afford the required devices to receive support virtually and/or due to the lack of culturally safe resources [8, 38, 39]. As a result, these barriers have adversely impacted help-seeking among underserved individuals affected by IPV during the pandemic. Therefore, it is important to develop policy measures to narrow the digital divide by allocating funds for increased access to digital technologies and reliable internet for underserved populations. It is also crucial to ensure sustainable funding for anti-violence organizations to provide culturally appropriate, holistic, trauma-and-violence-informed, and affordable virtually delivered IPV interventions.

Moreover, our findings show that determining the factors which constitute a safe, equitable, and accessible virtually delivered intervention for these population groups is complex and multifaceted, and requires collaboration among multiple levels of the social, health and political systems. It will be difficult to assure equitable and safe access to these interventions without addressing underlying factors of systemic discrimination and social exclusion that intersect on the axes of gender, race, ethnicity, ability, and geographical location.

## Data Availability

The datasets generated and/or analyzed during the current study are not publicly available due to potential privacy violation reasons but are available from the corresponding author on reasonable request.

## Abbreviations

IPV: Intimate Partner Violence
WHO: World Health Organization
EVAC: Ending Violence Association of Canada
PTSD: Post-Traumatic Stress Disorder
CPT: Cognitive Processing Therapy
CBT: Cognitive Behavioral Therapy
GBV: Gender-Based Violence
CBPR: Community-Based Participatory Research
TVI: Trauma- and Violence-Informed Approaches
